# Innovation in gastrointestinal surgery: the evolution of minimally invasive surgery—a narrative review

**DOI:** 10.3389/fsurg.2023.1193486

**Published:** 2023-05-23

**Authors:** Josephine Walshaw, Bright Huo, Adam McClean, Samantha Gajos, Jing Yi Kwan, James Tomlinson, Chandra Shekhar Biyani, Safaa Dimashki, Ian Chetter, Marina Yiasemidou

**Affiliations:** ^1^ Academic Vascular Surgical Unit, Hull University Teaching Hospitals NHS Trust, Hull, United Kingdom; ^2^ Faculty of Medicine, Dalhousie University, Halifax, NS, Canada; ^3^ Department of General Surgery, Bradford Teaching Hospitals NHS Trust, Bradford, United Kingdom; ^4^ Emergency Medicine Department, York and Scarborough Teaching Hospitals NHS Foundation Trust, York, United Kingdom; ^5^ Department of Vascular Surgery, Leeds Teaching Hospitals NHS Trust, Leeds, United Kingdom; ^6^ Department of Spinal Surgery, Sheffield Teaching Hospitals, Sheffield, United Kingdom; ^7^ NIHR Academic Clinical Lecturer General Surgery, University of Hull, Hull, United Kingdom; ^8^ Hull York Medical School, University of York, York, United Kingdom

**Keywords:** laparoscopic, robotic, minimally invasive, colorectal (colon) cancer, rectal cancer

## Abstract

**Background:**

Minimally invasive (MI) surgery has revolutionised surgery, becoming the standard of care in many countries around the globe. Observed benefits over traditional open surgery include reduced pain, shorter hospital stay, and decreased recovery time. Gastrointestinal surgery in particular was an early adaptor to both laparoscopic and robotic surgery. Within this review, we provide a comprehensive overview of the evolution of minimally invasive gastrointestinal surgery and a critical outlook on the evidence surrounding its effectiveness and safety.

**Methods:**

A literature review was conducted to identify relevant articles for the topic of this review. The literature search was performed using Medical Subject Heading terms on PubMed. The methodology for evidence synthesis was in line with the four steps for narrative reviews outlined in current literature. The key words used were minimally invasive, robotic, laparoscopic colorectal, colon, rectal surgery.

**Conclusion:**

The introduction of minimally surgery has revolutionised patient care. Despite the evidence supporting this technique in gastrointestinal surgery, several controversies remain. Here we discuss some of them; the lack of high level evidence regarding the oncological outcomes of TaTME and lack of supporting evidence for robotic colorectalrectal surgery and upper GI surgery. These controversies open pathways for future research opportunities with RCTs focusing on comparing robotic to laparoscopic with different primary outcomes including ergonomics and surgeon comfort.

## Introduction

Minimally invasive (MI) surgery has revolutionised surgery, becoming the standard of care in many countries around the globe. Observed benefits over traditional open surgery include reduced pain, shorter hospital stay, and decreased recovery time (1). Gastrointestinal surgery in particular was an early adaptor to both laparoscopic and robotic surgery (1).

This narrative review was carried out in accordance with the four steps outlined by Demiris et al. (2). Within this review, we provide a comprehensive overview of the evolution of minimally invasive gastrointestinal surgery and a critical outlook on the evidence surrounding its effectiveness and safety.

### History of laparoscopic surgery: a brief timeline

One of the earliest documented instances of minimally invasive (MI) surgery was around 460–375BC, where Hippocrates used an apparatus with structural similarities to endoscopes to examine the rectum under direct vision (3). In 936–1013AD a natural light source was incorporated into early endoscopic tools by Albukasim (4). Whilst there were several changes in the years to come, it wasn't until the invention of the light bulb by Edison in 1,880 endoscopic instrumentation changed significantly (5).

George Kelling, a surgeon in Dresden, attempted the very first laparoscopy in 1901. The technique, named Koelioscopie, entailed inserting a cystoscope through a trocar into a dog's abdominal cavity and insufflating oxygen (5). Kelling reporting of 45 laparoscopies (5), generated worldwide interest in laparoscopic techniques, including at the John Hopkins Hospital, where in 1911, Bertram Bernheim introduced laparoscopy to the United States (6).

In 1924, Zollikofer decided to use carbon dioxide (CO2) instead of atmospheric air for pneumoperitoneum. The rationale was that CO2 reduced discomfort as it is absorbed more easily by the human body, and is less combustible than air, facilitating the use of heat (7).

The next milestone was in 1929 when German physician Heinz Kalk invented a new lens which allowed him to view internal organs obliquely. Combined with the dual trocar puncture technique he developed, he achieved improved organ visualisation and passage of instruments into the peritoneum. Kalk subsequently published over 21 papers reporting laparoscopic operations on patients (6, 8).

In 1938, Janos Veress invented a needle, to help induce pneumothoraces as a treatment for tuberculosis. This was a spring-loaded, blunt needle bearing a cover which sprung forward to conceal a sharp needle in response to alteration in pressure as it entered the pleural cavity. Today the Veress needle is used to induce pneumoperitoneum in the abdominal cavity (8).

At this point in time, increasing interest in laparoscopy brought about rapid advancements for both equipment and operational technique surgery in the next decades- The invention of the “cold light” illuminator with the use of fibreglass in 1952 by Fourestier, Gladiu and Valmiere, eased concerns as it eliminated the occurrence of intraperitoneal burns caused by previous light sources (9, 10).

By the 1960s, laparoscopic surgery was widely used in gynaecological surgery. Kurt Semm, a German gynaecologist, designed an automated insufflator to closely monitor intra-abdominal pressure, increasing the procedure's safety and disposing of the need for a syringe to establish pneumoperitoneum (11). Semm also introduced thermocoagulation in laparoscopy and popularised procedures such as laparoscopic omental adhesiolysis, tumour biopsy, uterine perforation repair, endometrial implant coagulation and bowel suturing (6, 8). He was the first surgeon to perform a laparoscopic appendicectomy in 1983 (12).

In 1986, technological advances allowed for the projection of video camera images onto video screens (8). A laparoscopic cholecystectomy performed by Phillipe Mouret in 1987 was considered to be the first procedure during which this technology was used (10).

### Laparoscopic colorectal surgery

Jacobs et al. (13) performed the first laparoscopic-assisted colectomy in 1991. This was significantly more technically challenging compared to other MI operations performed around the same time period.

MI colorectal surgery was initially reserved for benign disease due to reported high port site seeding (21%) in colorectal cancer resections (14). This concern was later refuted with high-quality studies which demonstrated a rate comparable to open surgery in the area of 0.6–1.1% (15–18). Landmark randomised controlled trials (RCTs) were therefore designed to compare the oncological results of open vs. laparoscopic colorectal surgery (19, 20). In particular, the UK multicentre CLASICC trial (20) demonstrated similar short-term outcomes of 30-day mortality, lymph-node harvest, and oncological clearance as well as a long-term outcome of 10-year recurrence rates when comparing laparoscopic assisted to open groups (21). Further trials and meta-analyses demonstrated similar conclusions (21–27), providing evidence that laparoscopic surgery was feasible and safe.

It is to be noted that transverse colon and rectal cancer cases were excluded from some of these trials (19, 22, 25), which limited the generalisability of the conclusion to these patient groups. The introduction of new surgical techniques such as Total Mesorectal Excision, inspired a number of studies to compare MI and open approaches for these groups (28–30). COLOR II assigned adult patients with cancer up to 15 cm from the anal verge to laparoscopic vs. open surgery and cautiously concluded that laparoscopic in selected patients with rectal cancer performed by skilled surgeons demonstrates similar safety and oncological results to that of open surgery and does provide enhanced recovery (28).

Another landmark trial was the COREAN trial, which focused on mid and low rectal cancers after neoadjuvant chemotherapy (29). It demonstrated similar disease-free survival outcomes, whilst the 10-year follow-up trial confirmed the long-term oncological safety of laparoscopic surgery in this patient population (30).

ALaCaRT (Australasian Laparoscopic Cancer of the Rectum) and ACOSOG Z6051 Randomized Controlled Trial (31, 32), failed to demonstrate non-inferiority of laparoscopic surgery compared to open rectal cancer surgery for completion of resection and disease free survival and recurrence respectively. Although these findings are often misinterpreted in the literature as demonstrating inferiority of laparoscopic surgery, the results are merely inconclusive (33). Nevertheless, the misinterpretation of the two RCTs did create some concern regarding about the oncological outcome of laparoscopic total mesenteric excision (laTME) (33).

### TaTME

Transanal TME was proposed to address concerns raised for laTME (34). This involved dual transabdominal and transanal/ bottom-up dissection, with the expectation that it will diminish the technical difficulty of TME in narrow male pelvises, in obese patients (35). Several studies have shown TaTME to be safe (36–43), however authors expressed concern regarding the quality of the evidence this judgement was based upon (44, 45). These concerns escalated to the Norwegian moratorium for the technique in 2020. This was based on the high complication and local recurrence rates reported after a national audit (45). This looked at 157 patients who underwent TaTME; local recurrence rate was 7·6 per cent, eight local recurrences were multifocal or extensive. Eleven of 131 patients with an anastomosis (8·4%) had an anastomotic leak compared with 56 of 1,230 (4·5%) in the Norwegian Gastrointestinal surgery registry (45). These concerns were echoed by ACPGBI in the UK, recommending a “pause for reflection” (46).

Subsequent systematic reviews, although based largely on non-randomised studies, showed similar short (47) and long term oncological, functional outcomes (48–50), quality of life (QoL) (49) and complications (47, 50).

### Laparoscopic upper gastrointestinal surgery

Since Mühe performed the first laparoscopic cholecystectomy in 1985 (11), the use of laparoscopic techniques has seen a rapid expansion in upper gastrointestinal (GI) surgery. Cholecystectomy is now one of the most frequently performed laparoscopic procedure worldwide (51). Meta-analyses have demonstrated laparoscopic cholecystectomy to be equivalent to both open (52) and mini-open (52, 53) techniques for operative outcomes, while reducing patients' post-operative hospital stay and recovery time.

Laparoscopic surgery for upper GI malignancy has been utilised since the early 1990s with ever increasing scope as operative techniques and laparoscopic technology improve (54). Staging laparoscopy has been demonstrated to be an effective tool in aiding treatment and decision making in a wealth of upper GI cancers, while remaining a low-risk operation for the patient (55).

The first laparoscopic gastrectomy for malignancy was reported by Kitano et al. (56) in 1994, using a laparoscopically assisted technique requiring a mini-laparotomy to perform the anastomosis. Advantages proposed for this included reduced post-operative pain, improved nutrition and return to normal intestinal function, and reduced pulmonary complications. While their subsequent RCT did demonstrate successes in blood loss and wound size (57), there was no significant difference in time to return to oral nutrition or hospital stay. Larger trials have since shown improved post-operative morbidity with laparoscopic assisted surgery while maintaining similar oncological outcomes (58). The largest of these trials, the KLASS-01 (59) demonstrated no significant difference in survival rates between open and laparoscopically treated gastric cancer across 1,416 patients. More recently, total laparoscopic gastrectomy has been shown to be a safe alternative to both laparoscopically assisted and open gastrectomy. The main barrier is operative difficulty in achieving successful reconstruction of the GI tract (60).

Open operative management of oesophageal cancer has been the standard of care worldwide, however is highly invasive with associated morbidity due to the use of a thoraco-abdominal approach (61). The first MI oesophagectomy (MIE) was reported in 1992 by Cushieri et al. (62) utilising a right thoracoscopic approach. In a 115 patient RCT, Biere et al. (63) demonstrated reduced pulmonary complications, blood loss, and hospital stay in the MI approach group. However, operative time was significantly increased, with 14% of cases requiring conversion to open surgery. The ROMIO (Randomised Oesophagectomy: MI or Open) trial is an ongoing RCT comparing MIE with open oesophagectomy, with 526 participants undergoing analysis for operative outcomes (49). While multiple surgical approaches exist within the MI umbrella, there is no consensus on the optimal approach (64, 65).

### Natural orifice transluminal endoscopic surgery and single port laparoscopic surgery

Natural orifice transluminal endoscopic surgery (NOTES) builds on the idea of MI surgery promoting scarless, completely non-invasive procedures that do not require any skin incision. The first appendicectomy without an incision of the skin was performed transgastrically by Reddy and Rao in 2004 (66) with the first NOTES cholecystectomy being performed by Marescaux et al. (67) as recently as 2007. Although some isolated human cases of NOTES have been performed, the development of this technique is still in its infancy and has not been accepted as a routine general surgery procedure at present.

A compromise between NOTES and traditional laparoscopic practice is SILS. 1997 saw the first ever single port laparoscopic surgery (SILS) laparoscopic cholecystectomy performed by Navarra et al. (68) in which they inserted 2 trocars into the umbilicus, bridged only by a small strand of fascia which was then divided to aid gallbladder removal. Unlike NOTES, SILS does not accomplish totally non-invasive surgery. SILS does however aim to further minimise invasiveness by making a single abdominal incision to perform an operation via only one access point (69). Research continues into perfecting the technique and establishing it as a new gold standard for various operative procedures.

The reports on colonic surgery NOTES are from experimental settings, clinical studies were not employed due to worrying levels of complications observed in non-clinical projects (70). Conversely, there was a high level of enthusiasm concerning single-port colonic surgery. However, a number of studies set out to assess the potential impact, showed no significant benefit compared to “traditional” laparoscopic surgery (71–73).

## Robotic surgery

Robotic surgery introduced three-dimensional vision output, instrumentation with a significantly higher degree of movement freedom compared to laparoscopic instruments. This came hand-to-hand with increased cost and use of rather sizable pieces of equipment (74, 75).

The Arthrobot was the first robot to assist in surgery in 1983, manipulating the position of the patient's leg on voice command in arthroscopic surgery (76). Following this, robotic-assisted surgical procedures gradually began to emerge. In 1985 the Programmable Universal Machine for Assembly (PUMA) was used to orient a needle for CT brain tumour biopsies in adults (77) and thalamus astrocytomas in children (63), procedures normally suffering errors from unavoidable hand tremors. Three short years later, the PROBOT was used to perform the first robotic-assisted transurethral prostate resection at Imperial London College (78). The precision of robotic-assisted surgery was later applied in orthopaedic surgery with the ROBODOC which was found to be more effective than human hands to hollow the femur in preparation for total hip arthroplasty, avoiding common complications (79).

The Automated Endoscopic System for Optimal Positioning (AESOP), a voice-activated camera assistant, was introduced in 1994 as the first FDA-approved laparoscopic camera holder. Using the AESOP, the ZEUS surgical system used two additional robotic arms and a control console, allowing the benefit of a more ergonomic position for the surgeon (80). Following this, ZEUS was introduced clinically, with notable success in harvesting left internal mammary arteries for coronary artery bypass grafts (81). In 2001, the Lindberg Operation took place where surgeons Jacques Marescauz and Michel Gagner successfully remotely completed a laparoscopic cholecystectomy between New York City, USA and Strasbourg, France using ZEUS (82). However, delays between the control and operating station are notable reasons as to why telesurgery does not have more widespread success.

The da Vinci Surgical System was launched in 1997 and became the first FDA-approved comprehensive robotic system for laparoscopic surgery in 2000, with widespread applications in a variety of surgical fields. This offered the same degree of freedom as the human arm and slowly moved the surgeon further from the patient (80, 83).

Robotically assisted surgery has found a role in many surgical specialities and has allowed for the possibility of fully automated surgical operations. The Smart Tissue Autonomous Robot (STAR), designed at John Hopkins University, performed the first autonomous intestinal anastomosis in 2022 on porcupines over a one week period (84). The results indicated that the automated system outperformed expert surgeons' and robot-assisted surgery in terms of both consistency and accuracy, demonstrating the intricacy of robotics and the potential future of robotic surgery.

### Robotic colorectal surgery

Robotic colorectal surgery is becoming increasingly more common due to benefits including dexterity and accessibility, particularly in lower rectal cancer. The first robotic colectomy was performed in 2002 (85). By 2004, D'Annibale et al. (86) reported 52 cases including 10 rectal cases, concluding that similar operative and post-operative results were achieved with robotic and laparoscopic techniques.

In 2006 the first 6 cases of robotic TME were documented (72). Rawlings et al. (73) in 2007 reported 17 robotic right hemicolectomies and 13 anterior resections, concluding that robotic surgery is feasible and safe. A similar outcome was reached by Spinoglio et al. (87) in 2008 who compared 50 robotic resections to 161 laparoscopic operations.

A systematic review in 2014 assessed robotic surgery for rectal cancer (88). According to this report, robotic surgery demonstrated prolonged operative time compared to laparoscopic surgery and no difference in blood loss and oncological effect (positive circumferential margins and number of retrieved lymph nodes). Conversion rates to open surgery were found to be smaller for robotic surgery. Additionally, the substantially higher cost of robotic surgery and the lack of evidence regarding long-term oncological and functional outcomes were highlighted. A second systematic review by Milone et al. showed the robotic approach to be better in achieving a complete TME. However, no randomised controlled trials were included in their analysis (89).

The multicentre ROLARR trial (90) randomised 471 patients with rectal adenocarcinoma to robotic-assisted and conventional laparoscopic surgery. The primary outcome was conversion to open laparotomy and robotic-assisted laparoscopic surgery was found to not significantly reduce the risk of that. There was no significant difference in intraoperative or postoperative complications, 30-day mortality, or circumferential margin positivity.

### Robotic upper gastrointestinal surgery

Robotic-assisted upper GI surgery is a rapidly advancing field, due to benefits including providing a high degree of instrument freedom and stabilising the surgeon's tremor. However the current evidence base does not yet fully support its widespread use or justify the associated expense (91).

In 1997, the first robotic cholecystectomy (RC) was performed, marking the first use of the da Vinci Surgical System (83, 92). The current standard of care for the removal of the gallbladder is laparoscopic cholecystectomy (93). A recent systematic review has shown low rates of complications and comparable post-operative outcomes for RC vs. laparoscopic in the elective setting (94). However more studies are needed to assess more complex gallbladder disease outcomes. Several studies have also demonstrated that RC is effective and safe for general surgeons as a tool for robotic surgery training (92, 95).

Robotic-assisted MI oesophagectomy (RAMIE) was introduced in 2003 as a safe and viable option for oesophagectomy. The ROBOT RCT (96) showed that RAMIE yielded comparable oncologic outcomes to open oesophagectomy, with superior rates of surgically related postoperative complications, lower median blood loss, improved functional recovery at postoperative day 14, and better quality of life at discharge and at 6 weeks post-discharge. Long-term survival analysis showed that overall and disease-free survival was comparable, supporting the use of robotic surgery in oesophageal cancer (97). Additionally, Yang et al. (98) showed that RAMIE yielded shorter operation time with improved lymph node dissection compared to MIE, with no difference in complications including vocal cord paralysis, anastomotic leak, pulmonary complications, blood loss, and conversion rate. Long-term survival data from this trial is currently awaited. Further, a systematic review supports the use of RAMIE showing comparable mortality and reduced morbidity rates, however, operative time was found to be longer in patients receiving RAMIE compared to MIE (99).

## Conclusion

The introduction of minimally surgery has revolutionised patient care. Despite the evidence supporting this technique in gastrointestinal surgery, several controversies remain. Here we discuss some of them; the lack of high level evidence regarding the oncological outcomes of TaTME and lack of supporting evidence for robotic colorectal surgery and upper GI surgery. These controversies open pathways for future research opportunities with RCTs focusing on comparing robotic to laparoscopic with different primary outcomes including ergonomics and surgeon comfort.
